# *Hint1* gene deficiency enhances the supraspinal nociceptive sensitivity in mice

**DOI:** 10.1002/brb3.496

**Published:** 2016-05-27

**Authors:** Fei Liu, Jing Ma, Peng Liu, Zheng Chu, Gang Lei, Xiao‐di Jia, Jia‐bei Wang, Yong‐hui Dang

**Affiliations:** ^1^ College of Medicine & ForensicsXi'an Jiaotong University Health Science CenterXi'an710061ShaanxiChina; ^2^ Affiliated Stomatology Hospital of Xi'an Jiaotong University Health Science CenterXi'an710004ShaanxiChina; ^3^ Key Laboratory of the Health Ministry for Forensic MedicineXi'an Jiaotong University Health Science CenterXi'an710061ShaanxiChina; ^4^ Key Laboratory of Forensic Medicine of Shaanxi ProvinceXi'an Jiaotong University Health Science CenterXi'an710061ShaanxiChina; ^5^ Department of Pharmaceutical SciencesUniversity of Maryland School of PharmacyBaltimoreMaryland21201

**Keywords:** Gender differences, haploinsufficiency, *Hint1*, mice, supraspinal nociceptive sensitivity

## Abstract

**Introduction:**

Previous studies have indicated a possible role of histidine triad nucleotide‐binding protein 1 (HINT1) on sustaining the regulatory crosstalk of N‐methyl‐D‐aspartate acid glutamate receptors (NMDARs) and G‐protein‐coupled receptors (GPCRs) such as the *μ*‐opioid receptor (MOR). Both receptors are present in the midbrain periaqueductal gray neurons, an area that plays a central role in the supraspinal antinociceptive process.

**Methods:**

In the present study, a battery of pain‐related behavioral experiments was applied to *Hint1* knockout, heterozygous and wild‐type mice. Both the male and female mice were investigated to assess the differences between genders.

**Results:**

*Hint1*−/− mice presented significant shorter latency at 50°C in both male and female in hot plate test while no significant difference was found in tail filck test. In Von Frey hairs test *Hint1*−/− mice were more sensitive than *Hint1*+/+ mice, presenting a lower withdrawal threshold and enhanced relative frequency of paw withdrawal. The average flinches and licking time of *Hint1*−/− mice were more than that of *Hint1*+/+ mice in formalin test.

**Conclusion:**

The absence of *Hint1* gene‐enhanced supraspinal nociceptive sensitivity in mice, including thermal, mechanical and inflammatory hyperalgesia. Meanwhile, there was no certain evidence indicating the haploinsufficiency and gender differences of *Hint1* gene in pain‐related behaviors.

## Introduction

Pain is a complicated and subjective phenomenon, which involves not only the nociceptive fibers but also the cognitive and emotional processing in the brain (Zhu et al. 2010). As the International Association for the Study of Pain have stated, pain is the unpleasant sensory experience caused by damage or potential damage to bodily tissues. Clinical studies have revealed sex differences in pain experience, that is, women present significantly more behaviors of pain anxiety and pain intensity than men (Rhudy and Williams 2005; Ramirez‐Maestre and Esteve 2014). Large amounts of literature have indicated that chronic pain is frequently accompanied by psychiatric comorbidity, especially anxiety and depression (Liu and Chen 2014; Li 2015). Similarly, many animal experiments have also demonstrated that pain and emotional disorders coexist and exacerbate each other (Yalcin et al. 2011; Low et al. 2012).

The histidine triad nucleotide‐binding protein 1 (HINT1) is widely present in many tissues such as the liver, kidney, brain, and stomach in humans, (Klein et al. 1998), and is also highly expressed throughout the central nervous system (CNS) in mice, including the olfactory system, pallium, hippocampus, and a portion of the thalamencephalon, mesencephalon, and medulla oblongata (Liu et al. 2008). The *Hint1* gene is located on chromosome 5q31.2 a region correlated with schizophrenia (Straub et al. 1997). Vawter et al. (2002) applied a microarray with 1127 genes to identify differentially expressed genes in the brain samples from patients with schizophrenia. They found that *Hint1* was one of the three genes that uniformly decreased in expression in the dorsolateral prefrontal cortex (DLPFC) of patients with schizophrenia. *Hint1* knockout (*Hint1*−/−) mice were easier to be emotionally aroused in aversive situations (Varadarajulu et al. 2011; Zhang et al. 2015).

Guang et al. (2004) proved that HINT1 specifically bound to the C terminus of human *μ*‐opioid receptor (MOR). HINT1 also decreased the desensitization and phosphorylation of human MOR, which played an important role in morphine tolerance. For the subsequent behavioral experiment, *Hint1*−/− mice displayed an elevation of basal threshold, higher morphine‐induced analgesia, and a greater extent of tolerance development to morphine than *Hint1*+/+ mice in the hot plate test (Guang et al. 2004). In addition, Jackson et al. (2012) found that male *Hint1*−/− mice were less susceptible to acute nicotine‐induced antinociception in the tail flick test, rather than the hot plate test. *Hint1*−/− mice also showed increased allodynia than their wild‐type (WT) littermates in the chronic constriction injury (CCI) test (Garzon et al. 2015). Nevertheless, a genetic association study containing 2294 patients with cancer pain failed to find significant associations between *Hint1* genes and the dose of opioid for moderate to severe pain (Klepstad et al. 2011). The controversial phenomenon is highly correlated with the intricate functions of HINT1 and suggests that the role of HINT1 in different types of pain remains unknown.

The objective of the present study was to explore whether the deficiency of *Hint1* gene would modify the peripheral pain sensitivity in three pain‐related models in mice. Male and female *Hint1* knockout (*Hint1*−/−), heterozygous (*Hint1*+/−), and WT mice were used to analyze the differences in sexes and genotyping. Tail flick latency test (TFT), hot plate test (HPT), Von Frey Hair test, and formalin test (FT) were applied to evaluate the thermal nociceptive thresholds, mechanical nociceptive thresholds, and inflammatory pain, respectively.

## Materials and Methods

### Animals

Animals were bred under standard animal housing conditions in 12‐h/12‐h light‐dark cycle (lights on at 7:00 A.M.) at a room temperature of 22 ± 1°C and 55 ± 5% humidity. Mice were given free access to water and food. The experimental protocols were approved by the Xi'an Jiaotong University Laboratory Animal Administration Committee. All efforts were made to minimize the number of animals used and their suffering.

The experiments were performed using both male and female *Hint1* knockout, heterozygous and wild‐type control mice of 10–12 weeks of age. The littermates were generated as previously described (Su et al. 2003). Breeding pairs were supplied by Dr Jia‐bei Wang from the University of Maryland (Baltimore, MD). The genotypes of *Hint1*+/+, +/−, and −/− mice were confirmed with the polymerase chain reaction (PCR) using the forward primer 5′‐GCC CCC TGT AAA GTG CAG AC‐3′ and the reverse primer 5′‐CGC CCC AGT TAG TTA GT CAG‐3′ to generate a 339 bp product from the wild‐type allele, and the forward primer 5′‐GCC TGA AGA ACG AGA TCA GC‐3′ and the reverse primer 5′‐CGC CCC AGT TAG TTA GT CAG‐3′ to generate a 285 bp product from the mutant allele using genomic DNA extracted by tail biopsies of *Hint1*+/+, +/−, and −/− mice.

For all behavioral experiments, the animals were allowed to habituate the testing environment for 3 days, during which noxious stimuli were not delivered to them. Before the actual testing, the animals were exposed for 15 min to the testing devices. All the behavioral experiments were conducted during 9:00 A.M. to 12:00 A.M. The genotype of the mouse was not revealed by the appearance and the investigators were blinded to the genotype.

### Assessment of thermal nociception

The thermal nociception was examined by the noxious heat‐evoked tail flick test (TFT) and hot plate test (HPT). The TFT was a modified version of the experimental design described previously (Zhu et al. 2010). Mice were gently positioned by hands on the apparatus, and a hot lamp was focused on the distal two‐thirds of the tail, respectively. The time from start to remove the tail from heat was defined as the tail flick latency, and was recorded by a photocell‐triggered timer. The average of three measurements was used, with a cut‐off time of 10 sec avoiding tissue damage. The HPT was performed in the consecutive 3 days at an ascending temperature of 46 ± 1°C, 50 ± 1°C, 54 ± 1°C. Mice were placed on the hot plate apparatus (RB‐200; Tme Technology, Cheng du, China) with a cut‐off time of 30 sec, and the response latency to either a hind‐paw lick or jump was recorded.

### Assessment of mechanical nociception

Von Frey hairs test was conducted to assess the mechanical nociception of mice (Zhu et al. 2010; Ma et al. 2015). Ten stimuli were made with each of a series of Von Frey hairs (North Coast Medical Inc, Morgan Hill, CA) comprising the first 11 monofilaments (0.008, 0.02, 0.04, 0.070, 0.16, 0.40, 0.60, 1.0, 1.4, 2.0, and 4.0 g). The test was performed as previously reported by Bourquin et al. (2006). The test was started with filament 0.008 g and the next stiffer monofilament was applied in turns. The positive response was determined by paw withdrawal occurring twice in the 10 applications. The monofilament that first evoked a positive response was defined as the threshold and no further monofilaments were applied. We measured the number of positive withdrawal responses in ascending order for each monofilament of the series. Relative frequency of paw withdrawal, the ratio of positive responses to the total 10 times, was calculated as another index to assess the mechanical sensitivity.

### Assessment of inflammatory pain behavior

Formalin injection into the rat hind‐paw would induce pain‐related behavior. A quantity of 50 *μ*L of 5% formalin was injected subcutaneously into the dorsal surface of the right hind paw with a microinjection. Immediately after the injection, the animal was placed in the open Plexiglas box (30 cm × 15 cm × 15 cm) which permitted observation. The number of spontaneous flinches and the accumulating time of licking the injected paw was calculated every 10 min, for 60 min in all.

### Statistics

Statistical analyses of tail flick latency, withdrawal threshold, and the areas under the curve of flinches and licking time in formalin test were performed using two‐way analysis of variance (ANOVA) with sex and genotype as the between‐subject factors. The relative frequency of paw withdrawal and spontaneous flinches and the licking time/10 min in formalin test were performed using repeated measures ANOVA with between‐subjects factors to analysis, the differences between genotypes and sexes. Significant results were further analyzed using the Dunnett‐t post hoc test. Here *P* values <0.05 were considered statistically significant.

## Results

### General characteristics and genotyping of mice

**Figure 1:**
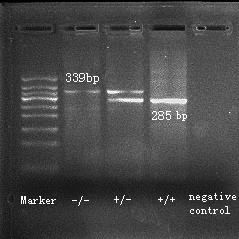
Genotyping of mice. Hint1−/− mice showed a 285 bp product, Hint1+/+ mice showed a 339 bp product and Hint1+/− mice showed both products.

The *Hint1*−/− mice did not present any obvious morphological or behavioral differences from the wild‐type mice. According to the results of PCR, *Hint1*−/− mice showed a 285 bp product, *Hint1*+/+ mice showed a 339 bp product and *Hint1*+/− mice showed both products (Fig. 1). The negative control was using distilled water rather than genomic DNA in procedure of PCR.

### Thermal nociception

**Figure 2:**
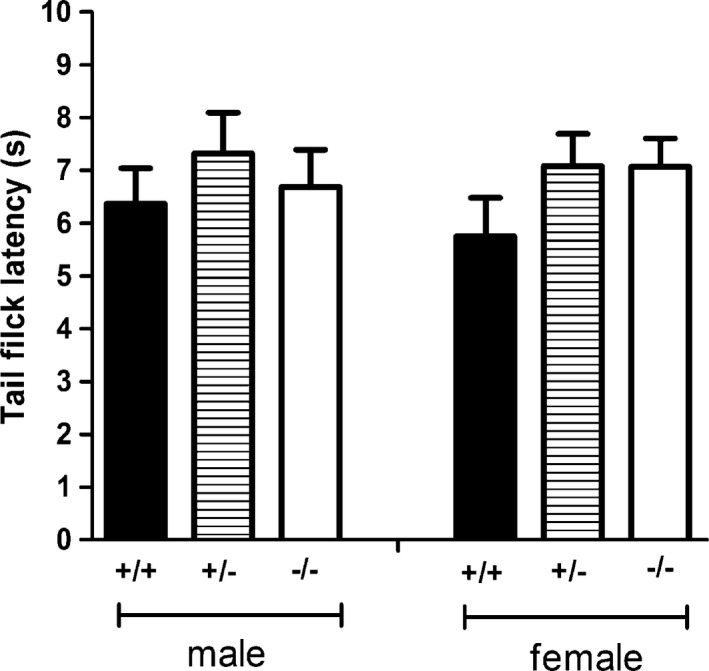
The tail flick latency of mice in tail flick test (*n *=* *8 of each group). There was no significant difference in both sexes and genotypes.

**Figure 3:**
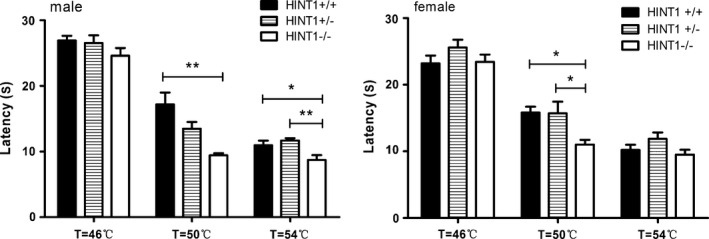
The response latency of mice in hot plate test (*n *=* *8 of each group). *Hint1*−/− mice presented significant shorter latency at 50°C in both male and female. The difference in sexes was not significant. *Denotes *P *<* *0.05 and **Denotes *P *<* *0.01, versus Hint1+/+ mice of the same sex.

The tail flick latency of *Hint1*−/− mice was shorter than *Hint1*+/+ mice, however, the difference in both genotypes and sexes was not significant in statistics (*F*(2, 44) = 1.587, *P *>* *0.05) (Fig. 2). In HPT, the temperature‐dependent latency was observed in all the groups, that is, as the temperature rose, the latency was curtate. *Hint1*−/− mice presented significant shorter latency at 50°C in both male and female (*F*(2, 44) = 14.012, *P *<* *0.05), and at 54°C in male mice (*F*(2, 44) = 10.906, *P *<* *0.05) (Fig. 3). The difference in sexes was not significant.

### Mechanical nociception

**Figure 4:**
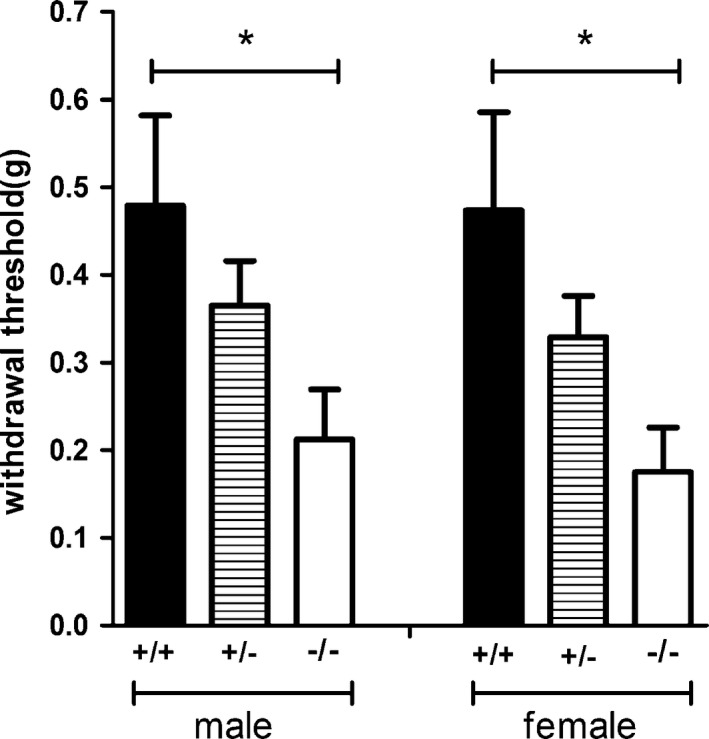
The withdrawal threshold of mice in Von Frey hairs test (*n *=* *8 of each group). *Hint1*−/− mice were more sensitive than *Hint1*+/+ mice, presenting a lower withdrawal threshold. The difference in sexes was not significant. *Denotes *P *<* *0.05 versus Hint1+/+ mice of the same sex.

In Von Frey hairs test *Hint1*−/− mice were more sensitive than *Hint1*+/+ mice, presenting a lower withdrawal threshold (*F*(2, 44) = 7.415, *P *<* *0.05) (Fig. 4). The difference in sexes was not significant.

**Figure 5:**
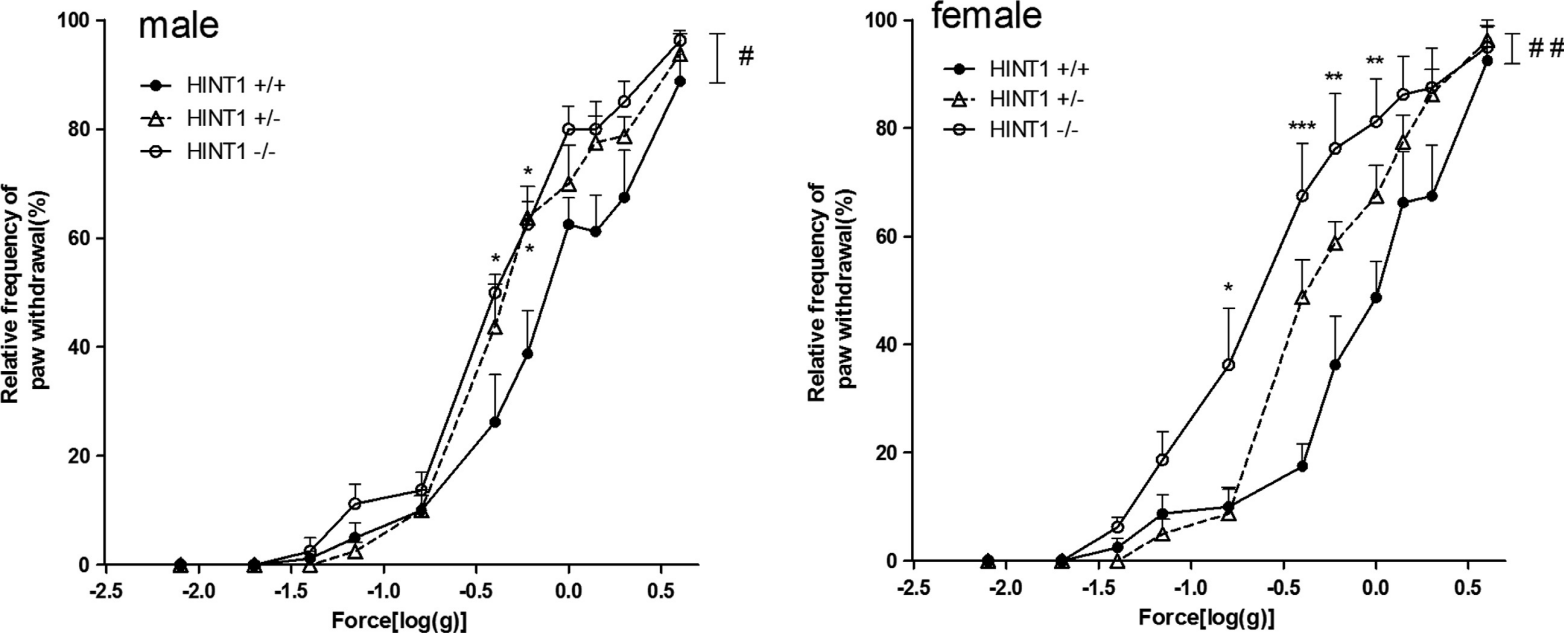
The relative frequency of paw withdrawal of mice in Von Frey hairs test (*n *=* *8 of each group). Male *Hint1*−/− mice exhibited mechanical pain‐like behavior compared with *Hint1*+/+ mice. The relative frequency of paw withdrawal of *Hint1*+/− mice was between those of the other two groups, but did not significantly differ from the *Hint1*+/+ mice. The mechanical pain‐like behavior was more obviously in female mice. *Denotes *P *<* *0.05 **Denotes *P *<* *0.01 ***Denotes *P *<* *0.001, compared with Hint1+/+ mice of the same sex at each point. ^#^Denotes *P *<* *0.05, ^##^Denotes *P *<* *0.01 between the groups of the same sex.

The relative frequency of paw withdrawal was calculated as follows. For male mice, *Hint1*−/− mice exhibited mechanical pain‐like behavior compared with *Hint1*+/+ mice (*F*(2, 44) = 5.005, *P *<* *0.05). The relative frequency of paw withdrawal of *Hint1*+/− mice was between those of the other two groups, but did not significantly differ from the control *Hint1*+/+ mice. The mechanical pain‐like behavior was more obviously in female mice (*F*(2, 44) = 6.345, *P *<* *0.01) (Fig. 5).

### Inflammatory pain behavior

**Figure 6:**
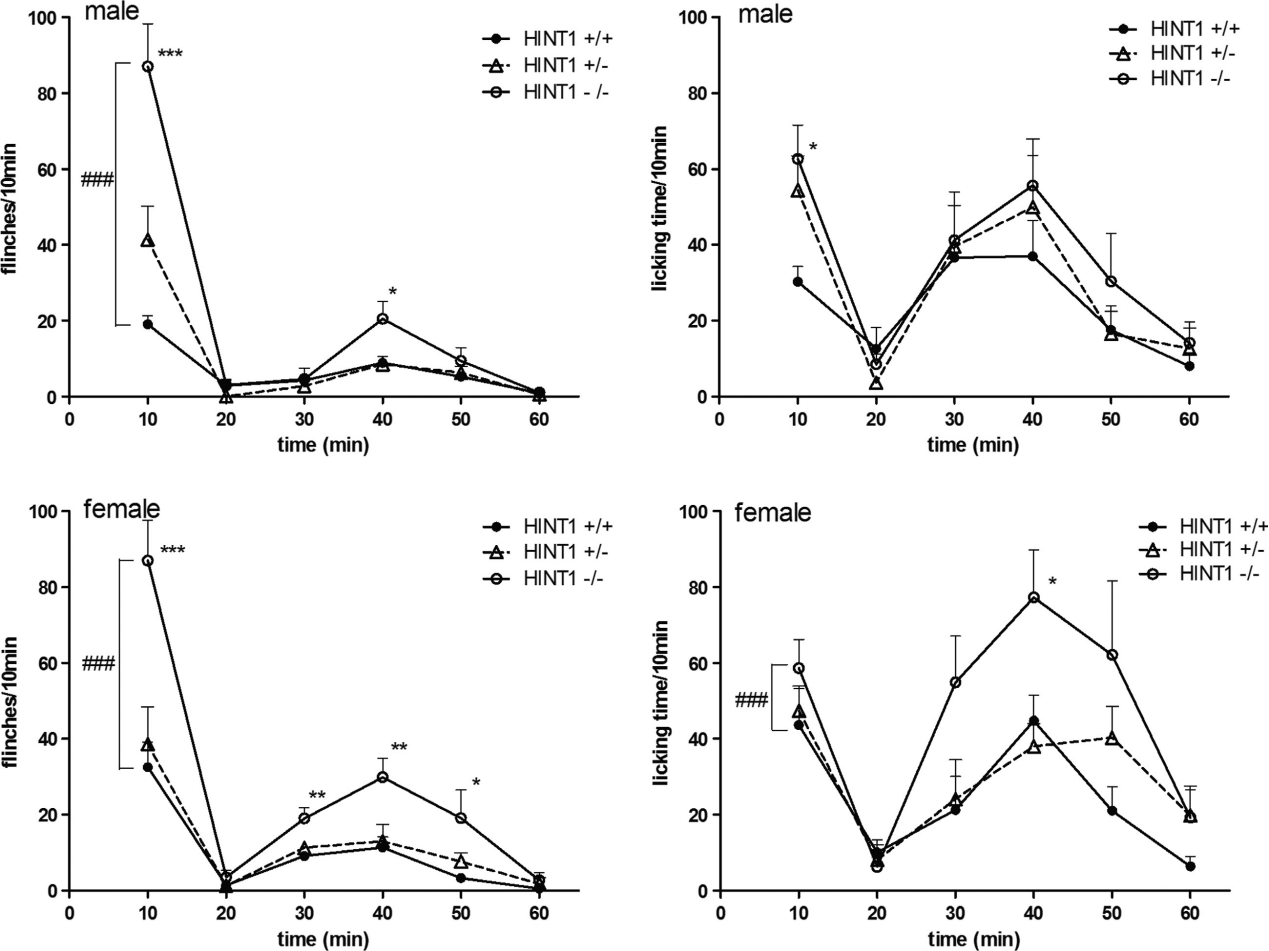
Spontaneous flinches and the licking time/10 min in formalin test (*n *=* *8 of each group). For the male, at time points of 10 min and 40 min, the average flinches of *Hint1*−/− mice were more than that of *Hint1*+/+ mice, as was licking time at 10 min. For the female, at time points of 10 min, 30 min, 40 min and 50 min, the average flinches of *Hint1*−/− mice were more than that of *Hint1*+/+ mice, as was licking time at 40 min. *Denotes *P *<* *0.05 **Denotes *P *<* *0.01 ***Denotes *P *<* *0.001, compared with Hint1+/+ mice of the same sex at each point. ^#^Denotes *P *<* *0.05, ^###^Denotes *P *<* *0.001 between the groups of the same sex.

**Figure 7:**
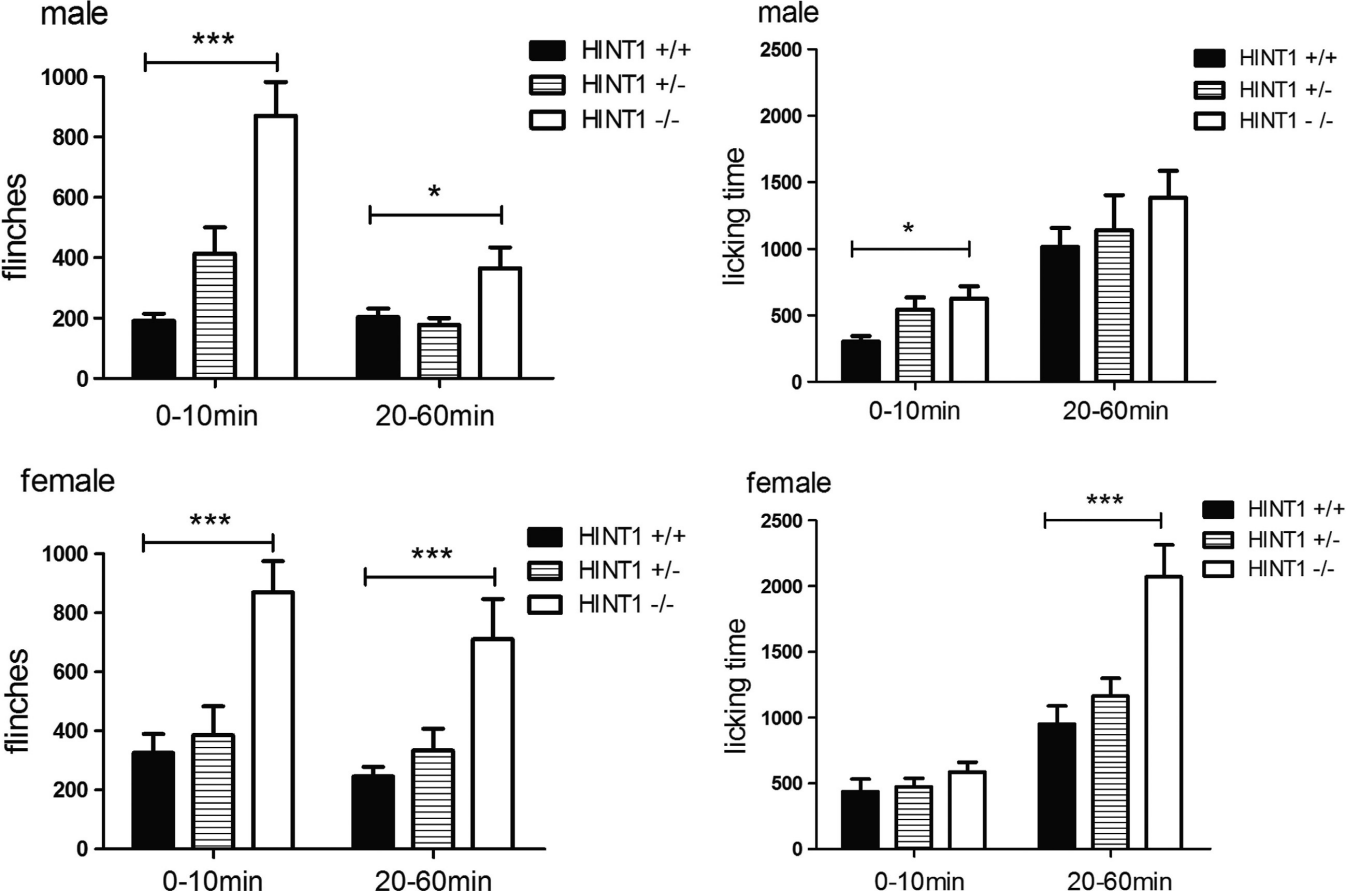
The areas under the curve of flinches and licking time in formalin test (*n *=* *8 of each group). In both phases, flinches were observed more frequently in *Hint1*−/− mice than in *Hint1*+/+ mice for male and female. Male mice showed more licking time in early phase and female showed more in the late phase. There was no significant difference between sexes. *Denotes *P *<* *0.05 ***Denotes *P *<* *0.001, compared with Hint1+/+ mice of the same sex.

A subcutaneous injection of formalin resulted in a typical biphasic display of flinches and accumulating licking time of the injected paw. For the male, at time points of 10 min and 40 min, the average flinches of *Hint1*−/− mice were more than that of *Hint1*+/+ mice, as was licking time at 10 min. For the female, at time points of 10 min, 30 min, 40 min and 50 min, the average flinches of *Hint1*−/− mice were more than that of *Hint1*+/+ mice, as was licking time at 40 min. Significant differences between genotypes of the same sex were observed in the flinches of male mice (*F* = 13.466, *P *<* *0.001), flinches and licking time of female mice (*F* = 27.962; *F* = 7.223, *P *<* *0.001) (Fig. 6). We divided the 1‐h test into two parts, an early‐phase (0–10 min) and a late‐phase (20–60 min), and calculated flinches and licking time by evaluating the areas under the curve. In both phases, flinches were observed more frequently in *Hint1*−/−mice than in *Hint1*+/+ mice for male and female (Fig. 7). Male mice showed more licking time in early phase and female showed more in late phase. However, there was no significant difference between sexes (*F*(1, 44) = 1.746, *P *>* *0.05).

## Discussion

In the present study, a battery of pain‐related behavioral experiments were applied to *Hint1*−/−, *Hint1*+/−, and *Hint1*+/+ mice to evaluate whether the deficiency of *Hint1* gene would modify the peripheral pain sensitivity. Both male and female mice were investigated to assess the sex differences. The major findings of this study were that the absence of *Hint 1* gene enhanced the supraspinal nociceptive sensitivity in mice, including thermal (in HPT but not TFT), mechanical, and inflammatory hyperalgesia. However, we did not discover that the pain regulation by *Hint1* showed sex difference.

Previous studies demonstrated that *Hint1* was involved in thermal nociception mechanism, the response varied depending on different drugs (Guang et al. 2004; Jackson et al. 2012). Thus, it is necessary to evaluate the basal thresholds in the absence of *Hint1* gene in different pain‐related models. Acute administration of morphine enhances antinociception of *Hint1*−/− mice in HPT (Guang et al. 2004). However, this effect is opposite for nicotine in TFT (Jackson et al. 2012), which may be due to the different cross‐regulation of HINT1 with *μ*‐opioid receptor or nicotinic acetylcholine receptors (nAChRs) (Guang et al. 2004; Jackson et al. 2010). In the present study, we observed that the response latency of *Hint1*−/− mice was significantly shorter than *Hint1*+/+ mice in HPT, while the three genotypes showed no significant differences in TFT. TFT mainly reflects spinal antinociceptive responses and HPT predominantly reflects supraspinal antinociceptive responses (Dennis et al. 1980; Ramabadran and Bansinath 1986). Our results suggest that *Hint1* gene plays different roles in the pain transduction via the spinal or supraspinal mechanism. To our knowledge regarding the physiological functions of HINT1 protein, the response latency of *Hint1*−/− mice is enhanced in HPT, which does not exactly match with our results (Guang et al. 2004). Controversial results were also found in an anxiety model of *Hint1*−/− mice (Barbier and Wang 2009; Varadarajulu et al. 2011; Jackson et al. 2012). These discrepancies are likely due to the differences in test settings, such as the apparatus, protocols, time of testing, and age of animals.

A considerable literature provides evidence that the *Hint1* gene is haploinsufficient as a tumor suppressor in mice (Su et al. 2003; Li et al. 2006). However, heterozygous mice were not included in most other studies focusing on the function of Hint1 gene or HINT1 protein. According to our results, not enough evidence demonstrates that the Hint1 gene is haploinsufficient. In some experiments, such as Von Frey hairs test and FT, the behavior of heterozygous mice was between that of knockout and wild‐type mice, which indicated that a certain amount of HINT1 protein might play an effective role, while in other experiments, heterozygous mice behaved more closely to knockout mice, which represent haploinsufficiency. Similar results were also found in the effect of chronic restrain stress on emotional behavior in Hint1−/− mice (Lian‐kang Sun, Peng Liu, Fei Liu, Zheng Chu, Jia‐bei Wang and Yong‐hui Dang, unpublished data). Meanwhile, in a study by Guang et al. (2004), there was no difference in HPT results between the heterozygous and wild‐type mice. Therefore, the function of the retained allele still needs further investigation using heterozygous mice.

Clinical and epidemiological research indicates that there are obvious sex differences in pain perception, pain‐related diseases, and analgesic effectiveness. Women exhibit higher incidence of chronic pains and lower tolerance to pain than men, which may be attributed to gonadal hormones, endogenous analgesic matter, and sociopsychological factors (Stoffel et al. 2005). This phenomenon is also found in rodents. Moreover, sex differences were also found in the effects of HINT1 on schizophrenia (Vawter et al. 2004; Chen et al. 2008), which may be due to the location of Hint1 gene on chromosome 5, which could also be affected by gonadal hormones. It is uncertain whether the sex‐specific association with this gene exists in pain‐related behaviors. However, we failed to discover notable differences between sexes in the present study. This might be related to the mouse strains, animal models, and types of experimental pain (Lacroix‐Fralish et al. 2005). For example, in lumbar radiculopathy model conducted by Lacroix‐Fralish et al. (2005), the most pain‐sensitive mouse strains following nerve root injury were 129P3/J, C58/J, and BALB/cJ, and the less pain‐sensitive strains were C57BL/6J, C3H/HeJ, and CBA/J. Meanwhile, female Sprague–Dawley and Long–Evans rats showed increased hypersensitivity compared to males, while no sex differences were observed in Holtzman rats (Lacroix‐Fralish et al. 2005).

As mentioned above, HINT1 protein is widely expressed in the CNS (Liu et al. 2008), and plays a vital role on sustaining the regulatory crosstalk between NMDARs and GPCRs (such as MOR) (Rodriguez‐Munoz et al. 2012; Sanchez‐Blazquez et al. 2013b). Both receptors are present in the midbrain periaqueductal grey neurons, an area that plays a central role in the supraspinal antinociceptive process (Garzon et al. 2015). The persistent overactivation of NMDARs has been correlated with the appearance of hypersensitivity to stimuli, chronic inflammatory pain, and neuropathic pain (Haley et al. 1990; Fisher et al. 2000; Mehta et al. 2012; Sanchez‐Blazquez et al. 2013a), while the hypoalgesia or antinociceptive effects have been observed when NMDARs were blocked (Ghalandari‐Shamami et al. 2011; Delawary et al. 2012). Interestingly, the expression of markers representing NMDAR function was increased in *Hint1*−/− mice compared to the *Hint1*+/+ controls (Makhinson et al. 1999; Rodriguez‐Munoz et al. 2012). In the present study, neuropathic pain was not involved which has been reported. In the CCI test of mice conducted by Garzon et al. (2015), the absence of *Hint1* gene increased allodynic responses in the ipsilateral nerve‐injured leg, and also in the contralateral paw compared with WT mice. However, the HINT enzymatic inhibitor significantly alleviated allodynia in wild‐type mice, but not in *Hint1*−/− mice. These different behavioral effects indicate that HINT1 enzymatic inhibition and the absence of HINT1 are not comparable (Guang et al. 2004; Rodriguez‐Munoz et al. 2011; Garzon et al. 2015). In Hint1+/+ mice, HINT1 participates in the adaptive changes of neuropathic pain. Even after the treatment of HINT enzymatic inhibitor, HINT1 protein sitll exists and associates with other signaling proteins. But this scaffolding role of HINT1 is completely eliminated in Hint1−/− mice, which motivates greater responses of NMDARs to direct stimulation and also an enhanced allodynia (Vicente‐Sanchez et al. 2013; Garzon et al. 2015). Thus, we speculate that the enhanced supraspinal nociceptive sensitivity (including thermal, mechanical, inflammatory, and neuropathic pain) of *Hint1*−/− mice might be due to the absence of HINT1, followed by the overactivation of NMDARs, which needs to be confirmed in future studies.

## Funds

The project was supported by National Natural Science Foundation of China (NSFC No. 81171262, 81371473).

## Conflict of Interest

We declare that there are no conflicts of interest.
